# Impact of Adding Polysaccharides on the Stability of Egg Yolk/Fish Oil Emulsions under Accelerated Shelf-Life Conditions

**DOI:** 10.3390/molecules26134020

**Published:** 2021-06-30

**Authors:** Selene Yadira Gonzalez Toledo, Jianping Wu

**Affiliations:** 4-10 Ag/For Centre, Department of Agricultural, Food and Nutritional Science, University of Alberta, Edmonton, AB T6G 2P5, Canada; selene.yg@gmail.com

**Keywords:** egg yolk, gum guar, gum arabic, high-pressure homogenization, oxidation, EPA, DHA

## Abstract

Polysaccharides can form interfacial complexes with proteins to form emulsions with enhanced stability. We assessed the effect of adding gum guar or gum arabic to egg yolk/fish oil emulsions. The emulsions were produced using simple or high-pressure homogenization, stored for up to 10 days at 45 °C, and characterized for their particle size and distribution, viscosity, encapsulation efficiency, oxidative stability, and cytotoxicity. Emulsions containing gum guar and/or triglycerides had the highest viscosity. There was no significant difference in the encapsulation efficiency of emulsions regardless of the polysaccharide used. However, emulsions containing gum arabic displayed a bridging flocculation effect, resulting in less stability over time compared to those using gum guar. Emulsions produced using high-pressure homogenization displayed a narrower size distribution and higher stability. The formation of peroxides and propanal was lower in emulsions containing gum guar and was attributed to the surface oil. No significant toxicity toward Caco-2 cells was found from the emulsions over time. On the other hand, after 10 days of storage, nonencapsulated fish oil reduced the cell viability to about 80%. The results showed that gum guar can increase the particle stability of egg yolk/fish oil emulsions and decrease the oxidation rate of omega-3 fatty acids.

## 1. Introduction

Emulsions have great potential for use as the delivery systems of lipophilic bioactive compounds, mainly for applications in food and pharmaceutical products. The effectiveness of these delivery systems can be assessed by the degree of protection they offer to the encapsulated compounds, or core material, from environmental stresses, such as those encountered during food processing and later in the gastrointestinal tract. 

A critical factor that will determine this effectiveness is the encapsulating material. Emulsions are commonly prepared using synthetic or natural emulsifiers (biopolymers) that can form a single layer surrounding the core material [1,2,3]. However, producing double-layer emulsion systems has been gaining attraction due to their potentially enhanced stability when compared with conventional monolayer emulsions [4,5,6,7,8]. In these systems, the primary coating is usually achieved with proteins, whereas the secondary layer can be formed using an oppositely charged polysaccharide [4,7]. The predominant stabilizing mechanism of polysaccharides is known to be steric, which, unlike proteins that generate electrostatic forces, forms emulsions with better stability under different environmental stresses. Thus, the addition of polysaccharides to a protein monolayer emulsion leads to the formation of interfacial complexes that reduce the van der Waals attractions between proteins and increase the steric and electrostatic repulsion between droplets, which could result in increased physical stability of the emulsion [9]. 

Polysaccharides have been extensively used in the food industry not only as thickening and gelling agents but also for their emulsifying properties. For instance, gum arabic, a polysaccharide derived from the exudate of the *Acacia senegal* tree, has been extensively used as an emulsifier for food applications [5,10,11]. The emulsifying properties of gum arabic are conferred by its amphiphilic nature: its hydrophobic side, the polypeptide chain, anchors the molecules to the lipid phase, while the arabinogalactan fraction (hydrophilic side) extends into the aqueous phase. Alternatively, guar gum, a linear nonionic polysaccharide, which consists of a chain of β(1–4)-linked mannopyranosyl units with an α(1–6)-linked D-galactopyranosyl chain every second residue [12], is commonly used in the food industry as a thickening and stabilizing agent. Resistance to pH changes, ionic strength, and/or harsh temperatures, such as heating and freezing, non-toxicity, and easy digestion, are among the potential advantages of adding polysaccharides as secondary layers for food applications [6,13]. Those characteristics have practical significance for encapsulation and preservation during processing and storage, as well as the targeted delivery of highly sensitive or unstable bioactive compounds, such as polyunsaturated fatty acids. 

Another important consideration during the production of emulsions is the processing technology selected, which can vary from low to high energy, such as spontaneous emulsification, high-shear mixing [4], high-pressure homogenization [14], microfluidization [2,3], ultrasonication, and membrane and microchannel emulsification. High-pressure homogenization has been widely used to form mono- and double-layer emulsions. The high shear force generated during the homogenization process entails the rapid movement of the encapsulating materials to the interfacial region and causes violent disruption of the droplets, making most droplets in the nano-size range (<200 nm) [15].

In this study, we hypothesized that the inclusion of polysaccharides in egg yolk/fish oil emulsions can produce emulsions with better stability. Therefore, we assessed the effect of adding gum guar or gum arabic on the stability and cytotoxicity of egg yolk/fish oil emulsions. The performances of two processing conditions, simple and high-pressure homogenization, were also evaluated. 

## 2. Results

### 2.1. Statistical Significance and Interactions between the Factors

A significance level of α = 0.05 was used for all statistical analyses. The influence of the fixed factors on the physicochemical characteristics of the main plots (egg yolk/polysaccharide emulsions) was analyzed using the package “agricolae” in R [16]. Differences between the main plots over time were analyzed using a linear model. The analysis of variance showed a highly significant (*p* < 0.001) influence of treatment, and the interaction treatment × time on the average particle size, encapsulation efficiency, and toxicity of the emulsions. The type of polysaccharide used as a secondary layer showed a significant influence (*p* < 0.01) on all the variables evaluated. On the other hand, time showed its highest influence (*p* < 0.05) only during interactions with factors. The effect of processing on the response variables was highly significant (*p* < 0.001). Finally, the highest influence (*p* < 0.001) of esterification type and concentration and their interaction with other factors was observed on the average particle size.

### 2.2. Treatment-Dependent Viscosity

**Figure 1:**
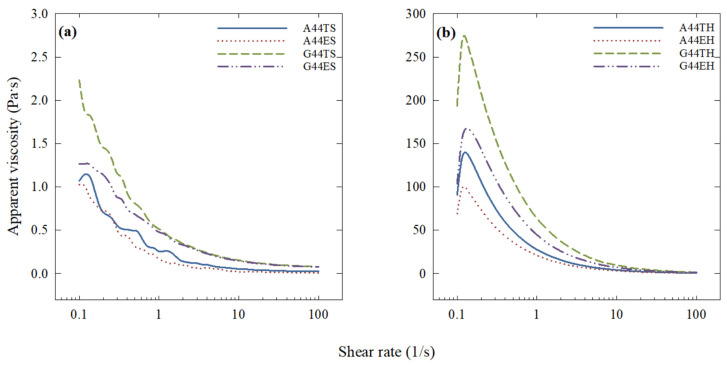
Effect of (**a**) primary (S) and (**b**) secondary (H) homogenization on the apparent viscosity of egg yolk/fish oil triglyceride (T) or ethyl ester (E) emulsions containing gum arabic (A) or gum guar (G). Each curve is the average of three replications. The viscosity of emulsions formed using secondary homogenization was significantly higher (*p* < 0.001) than those prepared under primary homogenization conditions. G44TH was significantly more viscous (*p* < 0.05) than all other treatments under the same processing conditions.

In this study, a significant effect of the polysaccharide type, processing, and esterification of fish oil on the apparent viscosity of emulsions was observed. The effect of simple (24,000 rpm, 4 min) and high-pressure (200 MPa) homogenization on the viscosity curves of samples containing 44% EPA + DHA is displayed in Figure 1a,b. Secondary homogenization had a highly significant influence (*p* < 0.001) on the apparent viscosity of all emulsions, regardless of the esterification type. Nevertheless, the esterification type of the fatty acids in the fish oil had a significant influence (*p* < 0.05) on samples within the same processing type. For instance, samples containing triglycerides had a higher viscosity than those containing ethyl esters. Overall, emulsions where gum guar was used as the secondary layer had higher viscosity (*p* < 0.05) than those using gum arabic.

### 2.3. Particle Size and Distribution as Affected by Treatment and Time

#### 2.3.1. Effect of Treatment and Time on the Particle Size Distribution

**Figure 2:**
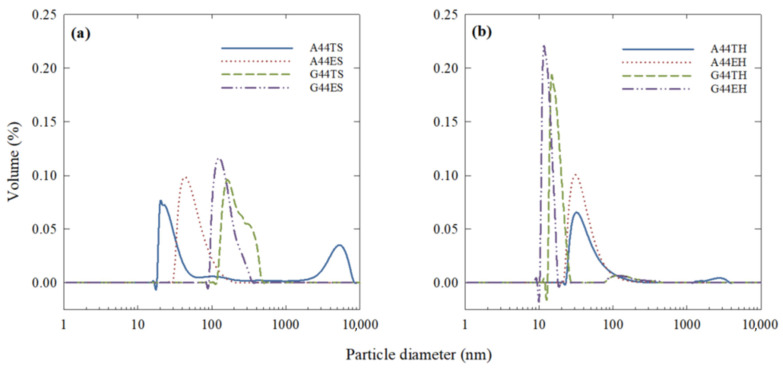
Effect of (**a**) primary (S) and (**b**) secondary (H) homogenization on the particle size distribution of egg yolk/fish oil triglycerides (T) or ethyl esters (E) emulsions containing gum arabic (A) or gum guar (G). Each curve is the average of three replications. All treatments showed a monomodal particle size distribution in the nano-size region after the high-pressure homogenization, with the exception of A44TH, which showed some residual in the micro-size region.

The effect of processing type on the particle size distribution of the emulsions containing 44% EPA + DHA triglycerides or ethyl esters is shown in Figure 2a,b. The distribution curves of samples using gum guar as the secondary layer were narrower and skewed to the smaller size end after the secondary homogenization. Moreover, emulsions containing gum arabic and triglycerides processed using primary homogenization (A44TS) showed a second peak in the micro-size region that almost disappeared after secondary homogenization (A44TH). On the other hand, A44ES (gum arabic + ethyl esters after primary homogenization) did not show an obvious second peak but the curve became more skewed to the lower size end after the secondary homogenization.

#### 2.3.2. Effect of Treatment and Time on the Average Particle Size

The mean particle size of emulsions containing triglycerides was significantly larger than those with ethyl esters at all levels evaluated. The most significant effect of time on the average particle size was observed at day 10 and the treatments most affected by time were those containing gum arabic.

**Figure 3:**
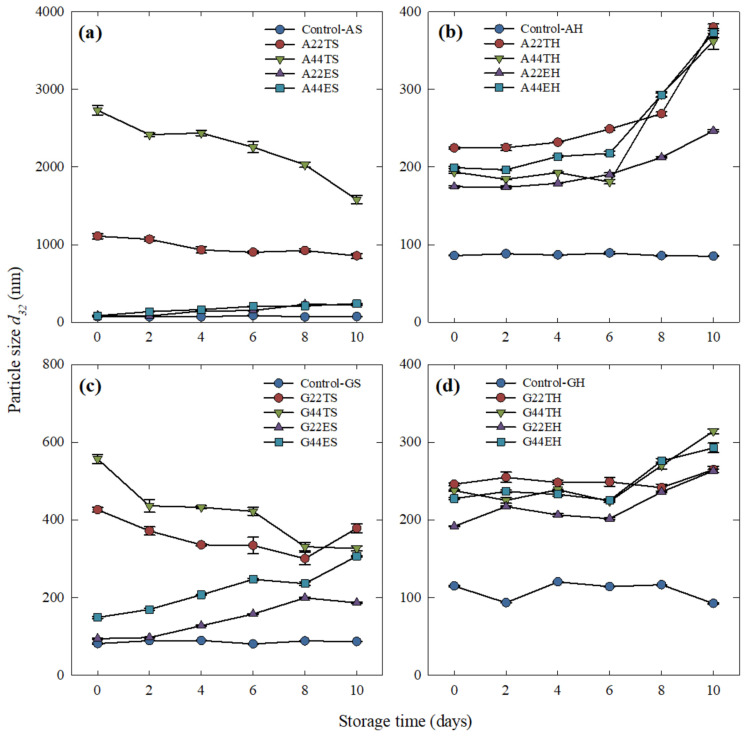
Average particle size of egg yolk/fish oil triglycerides (T) or ethyl esters (E) emulsions containing gum arabic (A) after (**a**) primary and (**b**) secondary homogenization, or gum guar (G) after (**c**) primary and (**d**) secondary homogenization. Changes in the particle size are shown over 10 days of storage at 45 °C. The graphs are organized to display differences between main plots (polysaccharide type, rows) and the influence of the split-plot (processing type, columns). Each data point corresponds to the mean ± standard error of three replications.

The effects of the processing conditions and time on the particle size of emulsions are shown in Figure 3a,b when gum arabic was used as a secondary layer and Figure 3c,d when gum guar was used. On day 0, the smallest average particle size corresponded to Control-AS (72.4 nm); however, this value was not significantly different from the other controls (-AH, -GS, and -GH). On the other hand, a significantly larger particle size was obtained in emulsions carrying triglycerides. Samples subjected to secondary homogenization (high-pressure homogenization at 200 MPa) showed a significantly smaller particle size (*p* < 0.01) than their counterparts processed only through primary homogenization. In our study, the strongest effect of high-pressure homogenization was observed in samples containing gum arabic and 22 or 44% triglycerides after secondary homogenization (A22TH and A44TH), which exhibited about 5- and 12-fold reductions in size compared to those formed using primary homogenization, A22TS and A44TS, respectively. A significant effect of storage on the particle size of emulsions stabilized by gum guar was observed only on day 10.

A significant effect (*p* < 0.05) of the EPA + DHA concentration on the particle size was also observed. At lower concentrations of the lipid phase, a smaller average particle size was recorded.

### 2.4. Influence of Treatment on the EPA + DHA Encapsulation Efficiency of Egg Yolk 

Table 1 shows the initial encapsulation efficiency and the total EPA + DHA that remained encapsulated after 10 days of storage at 45 °C for emulsions with the highest concentrations of EPA + DHA. On day 0, the encapsulation efficiency of emulsions containing gum guar and produced using secondary homogenization (G44TH and G44EH) was significantly higher than those using a combination of gum arabic and primary homogenization (A44ES and A44TS).

Differences in the efficiencies between the two polysaccharides were clearly observed after 10 days of storage when the analysis of variance showed a highly significant influence (*p* < 0.001) of treatment, time, and their interaction on the percentage of encapsulated oil, displaying a clear separation of the main plots. For instance, the four samples prepared with gum guar showed significantly higher (*p* < 0.05) encapsulation efficiencies than those of gum arabic. Furthermore, the effect of processing time was significant only in emulsions using gum arabic as the secondary layer after 10 days of storage, with those emulsions formed using secondary homogenization being the ones displaying the lowest release of the core material.

### 2.5. Formation of EPA + DHA Oxidation Products as an Indicator of the Emulsion Stability

**Figure 4:**
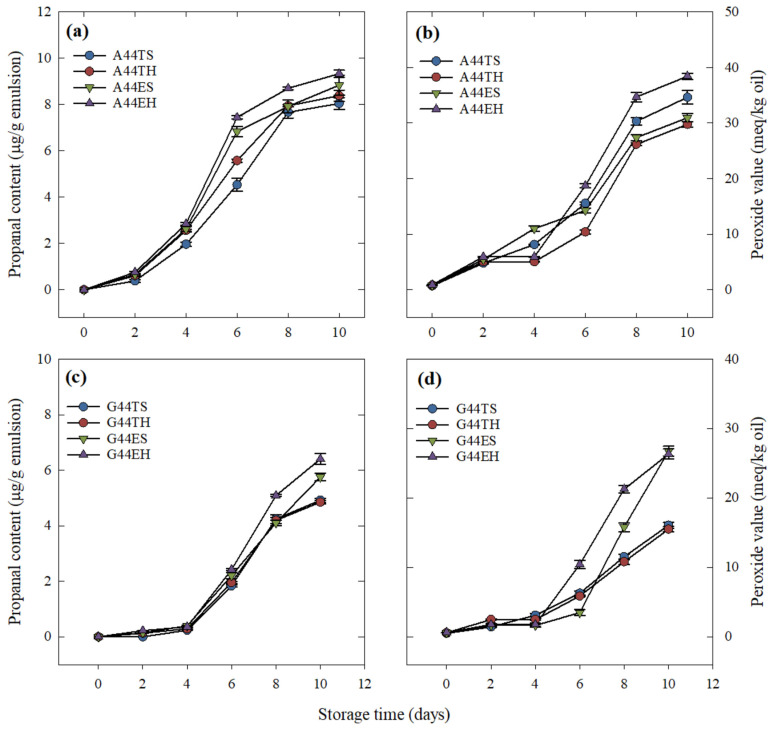
Oxidative stability of the emulsions over 10 days of storage at 45 °C. The graphs are organized to display differences between main plots (polysaccharide type, rows) and the influence of the split-plot (processing type, columns). (**a**) Propanal content of egg yolk/fish oil triglycerides (T) or ethyl esters (E) emulsions containing gum arabic (A) after primary (S) or secondary (H) homogenization. (**b**) Peroxide value of egg yolk/fish oil triglycerides or ethyl esters emulsions containing gum arabic after primary or secondary homogenization. (**c**) Propanal content of egg yolk/fish oil triglycerides or ethyl esters emulsions containing gum guar (G) after primary or secondary homogenization. (**d**) Peroxide value of egg yolk/fish oil triglycerides or ethyl esters emulsions containing gum guar after primary or secondary homogenization. Each data point corresponds to the mean ± standard error of three replications.

The propanal and peroxide formation in emulsions containing the highest concentration of EPA + DHA during storage at 45 °C is shown in Figure 4. The effect of the fixed factors on the oxidative stability of emulsions showed the same trend as their effect on encapsulated oil.

Emulsions using gum guar (Figure 4d) had higher stability, as shown by the lack of significant peroxide formation from day 0 to day 4 of storage, whereas those emulsions using gum arabic (Figure 4b) showed significantly higher values on day 2 of storage. Overall, a clear difference was observed between the main plots, with lower peroxide values for treatments using gum guar throughout the storage time evaluated. On day 10, emulsions containing triglycerides, regardless of the processing type, showed significantly lower peroxide values than those containing ethyl esters. 

A similar pattern was observed for propanal formation. No propanal formation was detected on freshly prepared emulsions (day 0), whereas at the end of the storage time (day 10), the lowest (α = 0.05) propanal contents, with values of 4.85 and 4.92 μg/g emulsion, were obtained from emulsions containing gum guar and triglycerides, G44TH and G44TS, respectively. The processing conditions did not show a significant effect on the oxidative stability of the emulsions over the evaluated storage period.

### 2.6. Toxicity of the Emulsions under Accelerated Shelf-Life Conditions on Caco-2 Cells

The toxicity of fresh emulsions (day 0) and the negative controls were evaluated at different concentrations (Supplementary Material) to screen for potential dose-dependent toxicity. No significant differences in cell viability were found within treatments at all levels evaluated. However, a lower variation was observed overall at a dose of 75 μg fish oil/mL medium; therefore, this concentration was used for the analyses over time.

**Figure 5:**
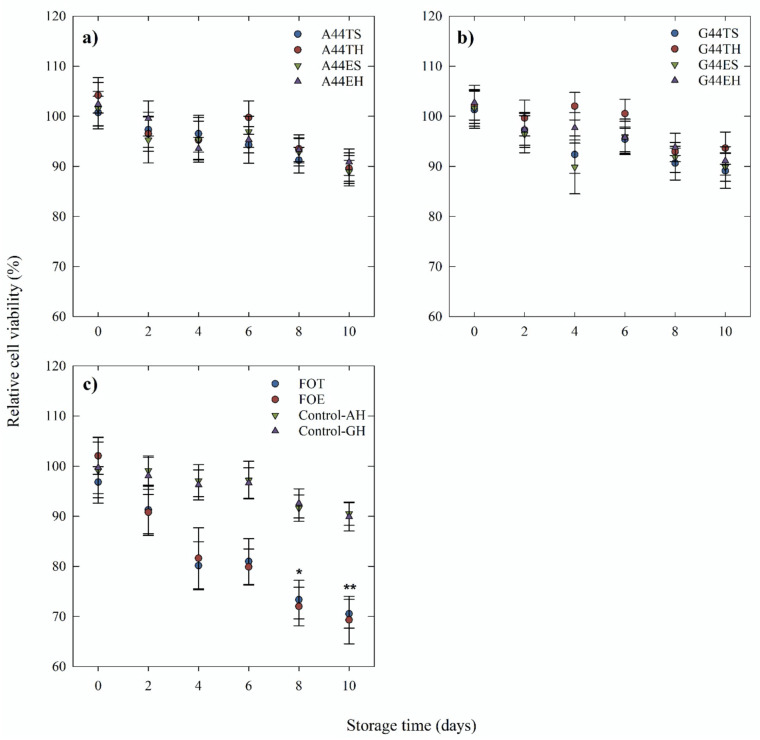
Toxicity of emulsions and nonencapsulated fish oil over 10 days of storage at 45 °C. (**a**) Relative viability of Caco-2 cells after treatment with egg yolk/fish oil triglycerides (T) or ethyl esters (E) emulsions containing gum arabic (A) produced by simple (S) or secondary (H) homogenization. (**b**) Relative viability of Caco-2 cells after treatment with egg yolk/fish oil triglycerides or ethyl esters emulsions containing gum guar (G) produced by simple or secondary homogenization. (**c**) Relative viability of Caco-2 cells after treatment with nonencapsulated fish oil triglycerides (FOT) or ethyl esters (FOE), and the control emulsions without fish oil (Control-AH, Control-GH) produced by secondary (H) homogenization. All samples were tested at a concentration of 75 μg fish oil/mL medium. Each point represents the mean of four to six replications ± standard error.

**Figure 6:**
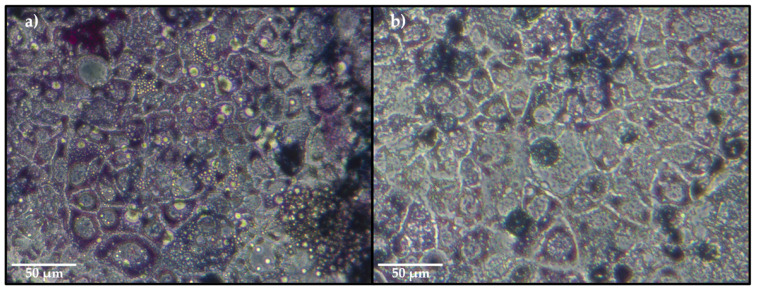
Light microscopy images displaying the purple formazan crystals resulting from the mitochondrial activity of Caco-2 cells after 24 h incubation with (**a**) egg yolk/fish oil ethyl esters emulsions containing gum guar (G44EH) or (**b**) nonencapsulated fish oil ethyl esters (FOE) on day 8 of storage.

The toxicity of the emulsions, the controls without fish oil, and nonencapsulated fish oil over 10 days of storage at 45 °C is shown in Figure 5. A significant decrease in cell viability was observed only for the negative control, resulting in an average viability of 69.52% for the nonencapsulated fish oil ethyl esters (FOE) (Figure 5c). This effect is clearly illustrated in the light microscopy images in Figure 6, showing the differences in the purple color intensity, which was related to the activity of mitochondrial dehydrogenases. 

## 3. Discussion

In this study, egg yolk was used as the primary emulsifier of fish oil triglycerides or ethyl esters. Gum guar or gum arabic was further added to the primary emulsion to act as a secondary layer. Simple or combined simple/high-pressure homogenization were the two homogenization processes tested. The resulting emulsions were developed to protect highly sensitive lipophilic bioactive compounds, namely, EPA and DHA, from environmental stresses. The effectiveness of the emulsions was given by their stability and their ability to prevent oxidation/degradation of EPA and DHA over 10 days of storage at 45 °C. 

It was shown that the rheological properties of oil-in-water emulsions are mostly dependent on the original properties of the biopolymers used as wall materials and their effective volume occupied in the emulsion system [3,4,17]. For instance, gum arabic is commonly used as an emulsifier in beverage industries due to its low viscosity, whereas gum guar is widely used in the food industry as a thickening agent. Gum guar belongs to the galactomannan family, which is known for its high-water-binding capacity that allows them to form viscous emulsions, even at low concentrations. Consequently, the emulsions where gum guar was used as a secondary layer had higher viscosity (*p* < 0.05) than those using gum arabic.

The particle sizes and distribution of an emulsion is an important characteristic to evaluate since it is an indicator of the efficiency of the emulsification process and the stability of the emulsions [4,18,19]. The narrower particle distribution observed in gum guar emulsions after secondary homogenization agreed with the observations of Bai et al. [2], who reported narrower monomodal particle distributions at higher homogenization pressures. On the other hand, the size distribution of emulsions containing gum arabic seemed to depend not only on the processing type but also on the esterification of the fish oil used as the core material. 

The overall changes in the distribution curves were also reflected in the average particle size of each treatment. In this case, the factor that caused the highest variability in the average particle size was the esterification type: triglycerides displayed a larger average size. In a previous study [14], we showed that during emulsification, the fish oil triglycerides have more affinity for the granule fraction of the egg yolk (micro-size region), thus increasing its volume percentage, which results in a larger average particle size. On the other hand, a high-pressure homogenization process was shown to form emulsions with smaller particle size, usually in the nano-size range, regardless of the biopolymer used as the coating material. For instance, high-pressure homogenization of sodium caseinate-coated fish oil emulsions at 12 kpsi showed an average particle diameter of 168 nm [7]; this trend was also observed in samples A22TH and A44TH. In comparison, the larger particle size obtained for their counterparts A22TS and A44TS could be the result of larger particles formed by a bridging flocculation effect [6,11]. These flocculated particles could be responsible for the peak observed in the micro-size region of the distribution curves (Figure 1a). Xu et al. [6] observed the same effect in emulsions using rice glutelin as a primary coating and 0.01% of anionic polysaccharides as the secondary layer. 

Polysaccharides can form thick interfacial layers, which can prevent droplet flocculation and coalescence [10,20]. Nevertheless, the bridging flocculation effect is observed if the concentration of polysaccharide molecules is not enough to cover the entire oil droplet in the case of single-layer emulsions or to bond with protein-coated droplets in double-layer emulsions, causing one molecule to adsorb to the surface of several droplets at the same time [4]. Bouyer et al. [11] suggested that this effect can also be related to the emulsification process in turbulent flow and storage. Compared to other surface-active biopolymers, gum arabic displays a lower affinity for oil-in-water interfaces; thus, a ratio as high as 1:1 gum arabic:oil phase ratio is needed to form emulsions that are more resistant to flocculation [12]. The observations from our study suggest that the concentration of gum arabic as a secondary layer was not high enough to prevent this effect; however, emulsions using gum guar at the same concentration did not show bridging flocculation. The effectiveness of gum guar, a nonionic polysaccharide, to prevent aggregation was the result of the hydration of its polar head groups. However, the accelerated shelf-life conditions tested in this study caused dehydration of these head groups that eventually resulted in aggregation [12], as observed by the increase in the average particle size. Hence, a significant effect of storage on the particle size of emulsions stabilized by gum guar was observed only on day 10. Shi et al. [18] also observed a significant increase of the particle size over the storage of fish oil-in-water emulsions stabilized by lecithin, gum arabic, or Tween 80. On the other hand, the resulting smaller particle size obtained at lower concentrations of EPA + DHA could be explained by the higher availability and faster adsorption of the biopolymer molecules used as emulsifiers to the oil phase surface due to the higher wall material:oil phase ratio [2], resulting in greater efficiency when coating the lipid droplets and forming smaller particles.

Another important characteristic to consider during the study of emulsions is their efficiency at preventing the release of the core material during processing and storage. Polysaccharides have shown potential to enhance the stability of oil-in-water emulsions using proteins as primary emulsifiers [6,13]. In these double-layer emulsions, proteins and polysaccharides can interact via covalent or noncovalent bonds, which influences the stability of the emulsions [9]. Therefore, this study assessed the differences between adding a neutral (gum guar) or an anionic (gum arabic) polysaccharide on the encapsulation efficiency and stability given by the total EPA+DHA that remained encapsulated during storage under accelerated shelf-life conditions (up to 10 days at 45 °C). 

The lack of significant differences in the encapsulation efficiencies between the two main plots in our experimental design (gum arabic versus gum guar) indicates that despite the bridging flocculation effect observed in treatments with gum arabic, the core material remained encapsulated within the continuous phase of the emulsion. Nevertheless, the clear differences between the efficiencies of the two polysaccharides to keep the EPA + DHA encapsulated after 10 days of storage could be partially attributed to the behavior of gum arabic under the pH conditions used during the processing of the emulsions. The initial egg yolk solutions were adjusted to pH 6.0 before proceeding with the primary homogenization. Under these conditions, the zeta potential of egg yolk is slightly negative [21], which could have caused repulsion between the gum arabic molecules and the proteins adsorbed at the surface of the lipid droplets, resulting in poor efficiency when forming a secondary layer [7]. Furthermore, as previously discussed, gum arabic requires a ratio of 1:1 (*w*/*w*) with the oil phase to reach its stable effect. Results from the encapsulation efficiency over time from this study seemed to be related to the overall particle stability found for the same treatments. The lack of significant differences between processing types on emulsions containing gum guar suggests that the stability of the emulsions at this stage was given mostly by the ability of the polysaccharide to act as an extra barrier to entrap the lipids in the core material and not due to the particle size of the emulsion. 

The encapsulation efficiency of an emulsion and its ability to prevent the core material release during processing and storage will impact its oxidative stability, as it is known that the nonencapsulated material will be more prone to oxidation in the presence of oxygen and high temperatures [18,22,23]. Therefore, a higher concentration of surface oil results in higher oxidation. This oxidation will adversely impact the nutritional value and sensory quality of the product [18]. Moreover, oxidation of long-chain polyunsaturated fatty acids, such as EPA and DHA, produces toxic compounds that are related to aging, mutagenesis, and carcinogenesis [24,25]. Thus, the prevention of lipid oxidation is of crucial importance in an oil-in-water emulsion system. The lack of significant effects of processing type on the oxidative stability of emulsions containing gum guar suggests that their stability was mostly the result of an efficient interaction between the egg yolk and the polysaccharide when acting as a barrier between the environment and the core material. 

The development of cytotoxic oxidation products from fish oil was studied to complete the assessment of the developed egg yolk/polysaccharide emulsions. For this purpose, the relative viability of Caco-2 cells was measured after incubation with fish oil from emulsions throughout storage and compared to that of cells incubated with nonencapsulated fish oil. Some compounds can display dose-dependent cytotoxicity on Caco-2 cells [26]; however, our emulsions did not show this effect at concentrations ranging from 50–100 μg fish oil/mL of incubation medium. Additionally, the lack of significant cell viability reduction of Caco-2 cells showed that our developed egg yolk/polysaccharide emulsions can prevent the formation of cytotoxic components from fish oil. Further studies are needed to evaluate the mechanisms with which these polysaccharides exert these beneficial properties.

## 4. Materials and Methods

### 4.1. Materials

White-shelled eggs were collected from the Alberta Poultry Research Centre (Edmonton, AB, Canada). Egg yolks were manually separated from the whites and rolled on Whatman no. 1 paper to eliminate the albumen residues. After puncturing the vitelline membrane, the egg yolk content was collected in a container placed in an ice bath and used to form the monolayer emulsions. 

Fish oil from Alaska Pollock (*Gadus chalcogrammus*) with a high content of eicosapentaenoic (EPA) and docosahexaenoic (DHA) fatty acids as triglycerides or ethyl esters was obtained from AlaskOmega Products (Organic Technologies, Coshocton, OH, USA). Caco-2 cells were obtained from the American Type Culture Collection and used from passages 19–32. Dulbecco’s modified Eagle’s medium (DMEM), fetal bovine serum (FBS), phosphate buffer saline (PBS), Hank’s balanced salt solution (HBSS), 0.25% (*w*/*v*) trypsin–0.53 mM EDTA, 1% non-essential amino acids, 1% (*w*/*v*) penicillin–streptomycin, and 4-(2-hydroxyethyl)-1-piperazineethanesulfonic acid (HEPES) were obtained from Gibco Invitrogen (Burlington, ON, Canada). Gum guar, gum arabic, an in vitro toxicology assay, and all reagents used for chemical analyses were obtained from Sigma-Aldrich (Oakville, ON, Canada). 

### 4.2. Emulsion Formation

**Figure 7:**
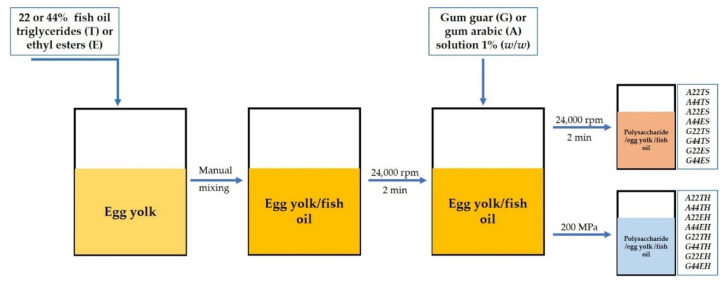
Graphical layout of the processes followed to prepare the emulsions and the sample codes for each group.

A graphical overview of the process followed to produce the emulsions is shown in Figure 7.

The moisture content of the freshly extracted egg yolk was determined using a convection oven set a 105–110 °C for 5 h. The 1% *w*/*w* polysaccharide solutions were prepared by stirring gum guar or gum arabic in cool Milli-Q water at 400 rpm until complete dissolution. The concentration and type of fish oil added followed the experimental design further described in Section 4.4. Egg yolk/polysaccharide emulsions were formed using two processing approaches: simple and high-pressure homogenization, as described below. 

#### 4.2.1. Simple (Primary) Homogenization

Fish oil was added to the egg yolk and mixed manually to facilitate its dispersion, then homogenized at 24,000 rpm for 2 min using a T25 Ultra Turrax (IKA Works Inc., Wilmington, DE, USA). The polysaccharide solution was then added to reach a final concentration of 0.25 g polysaccharide per 100 g of emulsion. Based on the egg yolk moisture content, the necessary amount of Milli-Q water was added to obtained 30% egg yolk solids in the final emulsion. This mixture was homogenized again at 24,000 rpm for 2 min. Thus, this ratio of 1:0.0083 egg yolk solids:polysaccharide was kept constant in all treatments, varying only the proportion of fish oil.

#### 4.2.2. Secondary (High-Pressure) Homogenization 

Egg yolk/fish oil mixtures were homogenized at 24,000 rpm for 2 min using a T25 Ultra Turrax. The corresponding polysaccharide solution was then added and mixed manually until homogeneous, keeping the 1:0083 egg yolk solids:polysaccharide ratio in the final emulsion. This mixture was subjected to secondary homogenization in a Pressure Cell Homogenizer FPG 12,800 (Stansted Fluid Power LTD, London, UK) at 200 MPa and a hydraulic relief pressure of 240 MPa.

#### 4.2.3. Accelerated Shelf-Life

To simulate accelerated shelf-life conditions, freshly prepared emulsions (simple and high-pressure) were transferred to air-tight sterile plastic tubes and stored at 45 °C for up to 10 days. Samples from the same experimental unit were analyzed right after preparation (day 0) and at days 2, 4, 6, 8, and 10. Based on preliminary results, all treatments were characterized according to their particle size and distribution over time, but only treatments displaying significant differences with the controls (mainly those containing 50% fish oil) were analyzed for viscosity, encapsulation efficiency, oxidative stability, and cytotoxicity. 

### 4.3. Characterization of the Emulsions

#### 4.3.1. Viscosity 

The apparent viscosity at 25 °C was determined using steady-state shear measurements (0.1–100 s^−1^) in a Modular Compact Rheometer 302 (Anton Paar, Graz, Austria). A concentric cylinder measuring tool with an active length of 120.2 mm and a positioning length of 72.5 mm was used to determine the viscosity curves of emulsions formed using primary homogenization. A 50 mm diameter parallel plate measuring tool and a 1 mm gap setting were used for the emulsions resulting from the secondary homogenization. 

#### 4.3.2. Particle Size and Distribution (PSD)

Emulsions were diluted 1:4 (*w*/*w*) with 1% (*w*/*w*) sodium dodecyl sulfate solution, then vortexed for 30 s prior to analysis to avoid multiple scattering. The 2 mL samples were placed in a disposable polystyrene cuvette for measurement. The particle size (as given by the Sauter mean diameter, *d*_32_) and distribution (given by the volume percentage) of the emulsions were measured via dynamic light scattering (DLS) in a Litesizer^TM^ 500 (Anton Paar, Graz, Austria) using backscattered angle (175 degrees) at 25 °C and 2 min of equilibration time. 

#### 4.3.3. Encapsulation Efficiency (EEf)

The encapsulation efficiency was calculated based on the concentration of EPA + DHA in the total and surface oil of the emulsions using the following formula:

EEf (%) = [(Total EPA + DHA − Surface EPA + DHA)/Total EPA + DHA] × 100(1)

Total oil was extracted following the chloroform:methanol method developed by Bligh and Dyer [27], whereas the surface oil extraction was conducted by washing with hexanes [22]. Both methods were modified to adapt to the matrix of the emulsions to obtain dependable yields according to previous observations [14]. 

Furthermore, the methylation and quantification of EPA + DHA were conducted following the method developed by Joseph and Ackman [28] using internal standard 23:0 methyl esters for triglycerides samples, and 23:0 ethyl esters for ethyl ester samples. The results are expressed as mg EPA + DHA/g oil.

The amount of surface oil obtained from most emulsions was below 25 mg; therefore, the weight of the oil was recorded for considerations in the final calculation. The appropriate internal standard was directly added into the tube containing the surface oil for further methylation. 

The EPA/DHA content was quantified after methylation by injecting 1 μL samples into an Agilent 7890 Gas Chromatograph with 5975 Mass Selective Detector using MSD Chemstation software. The carrier gas was Helium 5.0 and the column was an Agilent HP-5 MS with 30 m × 250 μm × 0.25 μm dimensions. The inlet temperature was set at 200 °C and operated in splitless mode. The oven was set to 50 °C for 5 min, then increased at 5 °C/min until 320 °C for 5 min. Total run time was 64 min. The mass spectrum was scanned from 40–500 *m*/*z*. 

#### 4.3.4. Propanal Content

The propanal formation in the emulsions, as assessed using headspace chromatography, was used as a marker for the oxidation of omega-3 fatty acids. About 2 g of the corresponding emulsion was placed into a 20 mL headspace vial, sealed, and heated at 60 °C for 30 min, followed by 30 min of equilibration time at room temperature. The headspace was then sampled manually using a Hamilton^®^ GASTIGHT syringe, 1725 N (Sigma-Aldrich, Oakville, ON, Canada). A total of 100 μL was manually injected in a capillary gas chromatograph equipped with a Restek Stabilwax (Crossbond Carbowax, Restek, Bellefonte, PA, USA) column measuring 20 m in length with a 0.53 mm internal diameter and 0.5 μm df while using helium as the carrier gas. The oven was set to 45 °C and the total run time was 2 min. The temperature of the detector and injector was 250 °C. The propanal content was calculated using a standard curve (r^2^ = 0.99) built with 0, 3, 6, 9, 12, and 15 μg propanal (98% purity) in Milli-Q water and expressed as μg propanal/g sample.

#### 4.3.5. Peroxide Value

The peroxide value (POV) was measured following the AOAC official method 965.33 [29]. The POV was expressed as meq peroxide per kg oil and calculated using the following formula:

meq peroxide/kg oil = (mL Na_2_S_2_O_3_ × molarity Na_2_S_2_O_3_ × 1000)/g test portion (2)

#### 4.3.6. Cytotoxicity Analysis

Caco-2 cells were routinely grown in supplemented DMEM, refreshing the medium every other day, and used for seeding at 80% confluence. The toxicity of emulsions was evaluated on days 0, 2, 4, 6, 8, and 10 of storage at 45 °C. The egg yolk/polysaccharide solutions without fish oil (controls) were tested under the same conditions to eliminate the effect of the carrier components. Non-encapsulated fish oil ethyl esters (FOE) were used as a negative control. 

The toxicity of the samples was assessed by measuring the relative viability of Caco-2 cells using the In Vitro Toxicology Assay Kit from Sigma-Aldrich, which is based on the MTT (3-[4,5-dimethylthiazol-2-yl]-2,5-diphenyl tetrazolium bromide) method developed by Mosmann [30]. 

Caco-2 cells were harvested with PBS containing 0.25% trypsin and 0.53 mM EDTA, then seeded in high-glucose and L-glutamine DMEM medium supplemented with 10% FBS, 1% non-essential amino acids, 1% penicillin and streptomycin, and 2.5% (*v*/*v*) HEPES to a concentration of 50,000 cells/mL. Then, 10,000 cells/well (200 μL) were placed on a 96-well plate and incubated at 37 °C in a humidified incubator with 5% CO_2_ for 24 h. 

After incubation, the original medium was replaced with the corresponding treatment suspended in the supplemented DMEM at final concentrations of 50, 75, and 100 μg EPA + DHA/mL medium and incubated for 24 h. The same concentration of EPA + DHA from FOE was used for comparisons with the negative control. Supplemented DMEM without emulsions was used as a blank reference for 100% viability. After the second incubation, the wells were gently washed 3 times with PBS. The cells were then incubated for 4 h with DMEM medium containing 10% MTT solution (5 mg/mL PBS). The medium was then removed and the formazan crystals formed on each well were observed at 40× in a Zeiss Axio Scope A1 light microscope (Zeiss, Oberkochen, Germany). The crystals were then dissolved by pipetting up and down with 200 μL of acidified isopropanol. The absorbance of each well was measured spectrophotometrically at a wavelength of 570 nm. The background absorbance of the plate was measured at 690 nm and subtracted from the value at 570 nm. This result was used to calculate the relative cell viability (%) using the following formula:

Relative cell viability (%) = (Abs treated cells × 100)/(Abs blank)(3)

### 4.4. Sample Identification and Statistical Analysis 

As an example, sample IDs were structured as follows: G44TS. The first letter refers to the polysaccharide type: G for gum guar or A for gum arabic. The following two digits refer to the percentage of total EPA + DHA from fish oil added, which was calculated based on the egg yolk’s dry matter. The third character indicates the esterification type: T for triglycerides and E for ethyl esters. Finally, the last letter refers to the process used for the emulsion formation: S means simple homogenization and H means high-pressure homogenization. Each group of treatments was paired with its control without fish oil, which were identified as Control-AS, Control-AH, Control-GS, and Control-GH, where the last two letters mean the same as specified before. Non-encapsulated fish oil ethyl esters (FOE) were used as negative controls for toxicity assays and oxidative stability. 

Emulsions were formed using a combination of polysaccharide type, processing method, concentration, and type of esterification of EPA + DHA. The experimental design was a split-split-plot in time. The main plots were the egg yolk:polysaccharide solutions. The fixed factor for the split-plot was the processing type at two levels: simple or high-pressure homogenization. Finally, the fixed factor for the split-split-plot was the type and concentration of fish oil at 4 levels: 22 and 44% EPA + DHA triglycerides and 22 and 44% EPA + DHA ethyl esters. Time (at 6 levels) was considered a fixed effect since its effect on the output variables was of interest. All chemical analyses were carried out in triplicates. Four replications were conducted for the toxicity analysis. Results were expressed as the mean and the standard error. A linear mixed-effects model was used to assess the significance of time as a factor and the fixed factors over time; a one-way analysis of variance (ANOVA) was used to find significant factors among treatments at a given time. Significant differences in the mean values were considered at *p* < 0.05 using Tukey’s test. All the statistical analyses were conducted using the statistical package R version 3.3.2 in R studio version 1.0.136 [13]. 

## Figures and Tables

**Table 1:** Encapsulation efficiency of emulsions containing 44% EPA + DHA (*w*/*w* egg yolk dry matter basis) and gum arabic (A) or gum guar (G) after primary (S) or secondary (H) homogenization and the remaining encapsulated oil after 10 days of storage at 45 °C. The percentages are given based on the total EPA + DHA quantification. Every value corresponds to the mean ± standard error of three replications. Different letters within columns mean significant differences between treatments. The encapsulated oil efficiency was significantly lower for all treatments at day 10. Significance was considered at *p* < 0.05.

Treatment	Encapsulation Efficiency (%) at Day 0	Encapsulated Oil (%) at Day 10
A44TS	97.3 ± 0.4 ^b^	80.7 ± 0.7 ^d^
A44TH	98.4 ± 0.2 ^ab^	89.7 ± 0.3 ^c^
A44ES	97.0 ± 0.2 ^b^	91.0 ± 0.7 ^c^
A44EH	98.4 ± 0.2 ^ab^	90.6 ± 0.5 ^c^
G44TS	98.0 ± 0.2 ^ab^	94.7 ± 0.2 ^a^
G44TH	99.5 ± 0.1 ^a^	94.6 ± 0.3 ^a^
G44ES	97.4 ± 0.1 ^b^	94.6 ± 0.4 ^a^
G44EH	99.4 ± 0.1 ^a^	92.8 ± 0.3 ^b^

## Data Availability

The data that support the findings of this study are available upon request from the corresponding author.

## Supplementary Materials

The following are available online, Figure S1: Toxicity of emulsions at day 0.

## Sample Availability

Due to the nature of the samples, they were not physically kept.
