# Supplementary material for: Impact of Adding Polysaccharides on the Stability of Egg Yolk/Fish Oil Emulsions under Accelerated Shelf-Life Conditions
Source: Molecules. 2021 Jun 30;26(13):4020. doi: 10.3390/molecules26134020 (PMC8271835; doi:10.3390/molecules26134020)
Supplement: Supplementary file 1 [file molecules-26-04020-s001.zip › molecules-1260965-supplementary.pdf]

Supplementary material

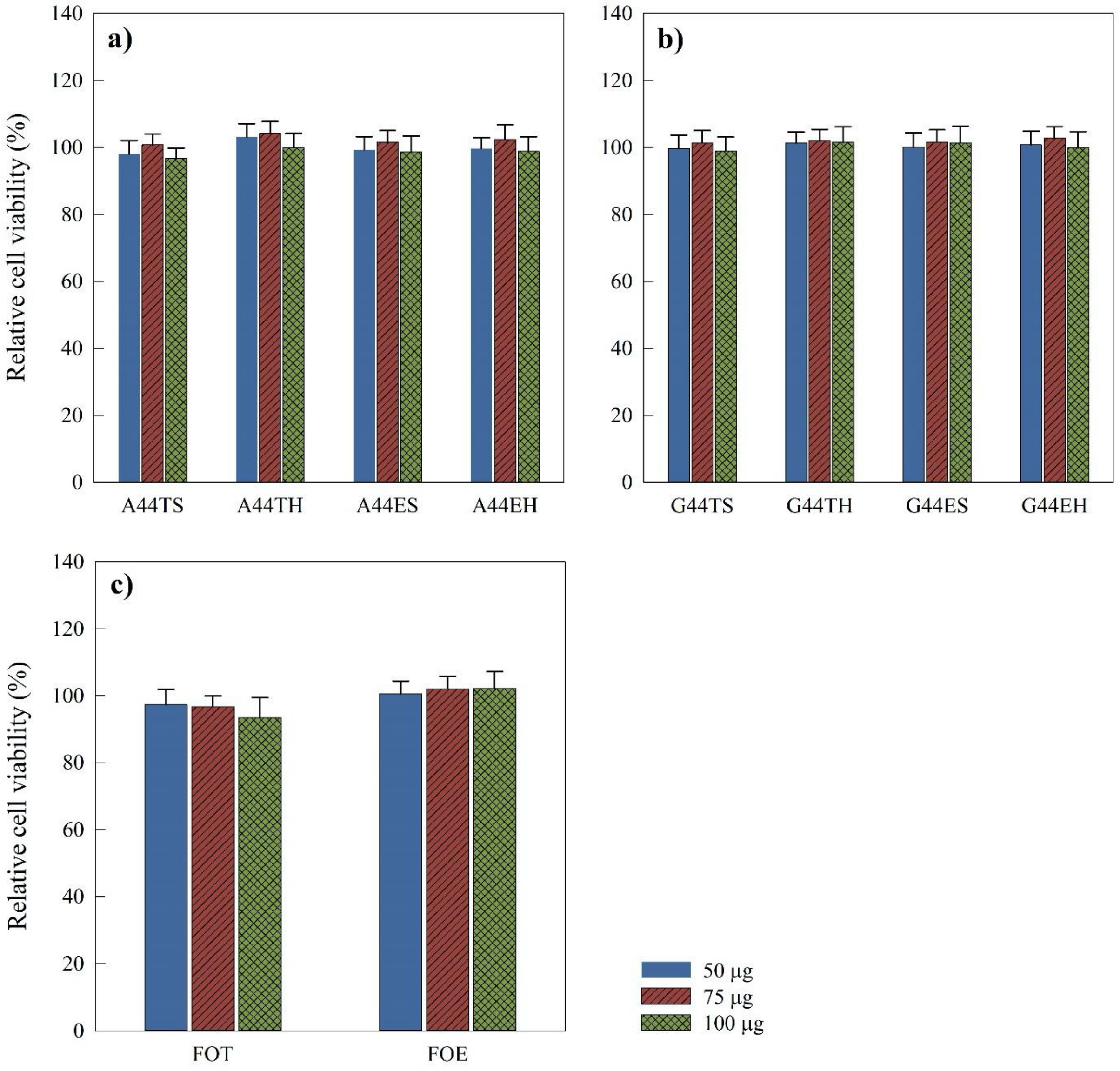

Figure S1. Toxicity of egg yolk/fish oil triglycerides (T) or ethyl esters (E) emulsions containing gum arabic (A) or gum guar (G) formed by primary (S) and secondary (H) homogenization, and the non-encapsulated fish oil triglycerides or ethyl esters controls (FOT and FOE, respectively) at 3 different concentrations (µg fish oil / mL medium) at day 0. Each bar represents the mean ± standard error of four to six replications. No significant differences with the blank (medium without emulsions) were found at $\alpha = 0.05$.
